# A Practical Treatise on the Management and Diseases of Children

**Published:** 1837-04-01

**Authors:** 


### A PRACTICAL TREATISE ON THE MANAGEMENT AND DISEASES OF CHILDREN. By *Richard T. Evanson*, M.D. Professor of Medicine, and *Henry Maunsell*, M.D. Professor of Midwifery in the Royal Coll. of Surgeons in Ireland. In one Volume 12mo. Dublin and London, Dec. 1836.

WE think there was ample field for a new treatise on the management and diseases of infants. The treatise of Underwood has long been antiquated, nor could all the notes and emendations of his editors make the edifice a sightly or consistent structure. The authors of the work before us have had the advantage of investigating the subject of infantile diseases conjointly in a public institution—an advantage which no private medical man, however extensive his practice, could probably have. The observations being made conjointly too, offer a greater guarantee of correctness and authenticity than if they emanated from a single source, however respectable. From their acquaintance, also, with foreign works, they have been able to bring up the anatomy, physiology, pathology, and even therapeutics, to a far higher level than is to be found in any previous work in the English language.

The volume is divided into 12 chapters, and each bears the signature of its author. The first, by Dr. Evanson, gives a very neat sketch of the anatomy, physiology, and growth of the infant, from birth up to the age of eight years. Upon this chapter we need not, of course, dwell. The second chapter is by Dr. Maunsell, and embraces the management and physical education of children. This chapter ought to be printed in gold letters, and hung up in the nursery of every family. It would save many lives, and prevent much suffering. We are tempted to make a short extract from this chapter, on the injurious effects of frequent mercurial purges, given to infants by mothers and nurses.

"And as the multitude has, in the case of the system in question, but one idea cognate with either remedy or preventative, viz. the idea of purging, the propriety of adopting a regular system of purgation with healthy children, has been admitted almost without question. There cannot, however, be a more dangerous absurdity than such a system; and in proof of our opinion, we shall ask a single question, and bring forward one fact. What is the mode of action and effect of every aperient drug in the pharmacopœia? Is it not irritation, direct or indirect, of the intestinal mucous membrane, in a greater or lesser degree; and does it not produce, as a necessary effect, in every instance, a larger or smaller quantity of increased secretion? So much for our question. We presume that no person, but a devoted disciple of St. John Long, will maintain that irritation, producing increase of secretion, can ever be necessary in the *healthy* body. With respect to our fact—let any person with regular, or moderately regular bowels, take an aperient, and he will certainly find that, after its immediate effects have passed away, a state of costiveness will remain. The tendency of nature to periodical movements, has been interrupted by the production of evacuations at irregular periods, and she requires some time to enable her to recur to the simplicity of her original design. We wish it to be distinctly understood, that what we are now advancing has no reference to the *use* of purgatives in disease, but solely to their *abuse* during health; and we are the more particular in endeavouring to convey our own views upon the subject, because we know that the impropriety of a needless resort to medicines of this kind, is not sufficiently thought of by medical men. We have, indeed, too often had occasion to lament over the display of drugs upon the mantel-piece of even a medical man's nursery, not to feel special interest in this matter. The least judicious would surely grieve, did he see an ointment of cantharides rubbed to a healthy skin, with no other apparent intention than that it might be followed by a healing salve; yet, what less absurd, is portended by the accumulation, in such a situation as we have alluded to, of packets of aperient draughts, bundles of restraining powders, and bottles of carminative mixture—all designed for the same unlucky children. This is no imaginary description, we have but too often seen its original; yet, better and more merciful would it be, towards many of the victims, to destroy them in the birth, than, by such a course, to provide for them the enduring miseries of an irritable intestinal mucous membrane." 71.

Upon the mental cultivation of infants, Dr. M. makes some judicious observations. The exact time for commencing the communication of scholastic knowledge cannot be easily determined. He conceives that it should not be begun before the sixth year. The practice, however, is very different among all classes of society. The child is often drilled and harassed with book-learning from four years upwards. We make no apology for introducing the following extract from the second edition of the "Economy of Health," by Dr. Johnson, on the same subject.

"I have alluded to the Spartan custom of leaving the youth, during the first seven years, under the guidance of the parents, who permitted the physical powers of their offspring to develop themselves without control. What is the case with us? During a considerable portion of that period the youth is 'got out of the way,' and imprisoned in a scholastic hot-bed or nursery, where the 'young ideas,' instead of being left to shoot out slowly, are *forced* out rapidly, to the great detriment of the intellectual soil, thus exhausted by too early and too frequent crops." 14.

"The first seven years of life must not be given up entirely to the physical development of the constitution; though that is a most important part of the parent's duty. A great deal of moral culture may be effected in this period: but I apprehend that it ought to be very different in kind, in mode, and in degree, from what it is at present. During several years of this first Septenniad, the children of the lower, and even of the middle classes, are cooped up in a crowded and unwholesome school-room, for many hours in the day, to the great detriment of their health and morals, and with very little benefit to their intellectual faculties. Among the higher classes, it is not so bad; yet there, the children are too much drilled by tutor or governess, and by far too little exercised in body.

The principle which I advocate is this: that during the first, and even during the second Septenniad, the amount of elementary learning *required* should be less, and the daily periods of study shorter;—that sport and exercise should be the regular and unfailing premium on *prompt and punctual* acquisition of the lessons prescribed—in short, that elementary education should be acquired 'cito, tuto, ac jucundè'—instead of being a wearisome task, irksome to the mind, and injurious to the body.

But if I declare myself adverse to the system of precocious exercise of the intellect, I am an advocate for early moral culture of the mind. It is during the first years of our existence that the foundation of *habits* and *manners* is laid; and these will be good or bad, afterwards, according to their foundations. ORDER is truly said to be 'Heaven's first law'—and so it should be the first injunction on childhood. The brightest talents are often rendered useless by the want of order and system in our amusements, studies, and avocations. The best temper or the purest intention will not compensate for want of regularity, industry, and punctuality. Habit is the result of *impression*, rather than of *reflection*; and youth is the age for receiving impressions, rather than for exercising the judgment. Order may be instilled into the juvenile mind, long before that mind is capable of perceiving the utility of the discipline; in the same way that the rules of grammar are learnt, before the application of these rules can be even imagined by the pupil. From long study, and, perhaps, a considerable knowledge of human nature, I most earnestly exhort parents, guardians, and tutors, to enforce, with all their energy, the most rigid system of order, regularity, and punctuality, from the very earliest period of infancy up to the age of discretion. Half, and more than half of our miseries, crimes, and misfortunes, in after life, are attributable to the misplaced indulgence, or culpable negligence of our parents. 'Spare the rod and spoil the child,' is a maxim that was founded in experience, though it has been nearly exploded by speculative philanthropists, not deeply versed in the knowledge of man. The rod, in most cases, may be spared; but, if order and obedience cannot be enforced by other means, the rod should be applied.

The whole material world, and, as far as we can judge, the whole universe, is subjected to, and governed by, certain laws of periodicity, which preserve order and harmony everywhere. Our mental and corporeal constitutions are controlled by similar laws of periodicity, and we should subject all our actions, passions, pleasures, and labours to laws, in imitation of those which Nature has established. Thus in infancy and youth, the sleep, exercise, play, meals—every thing, in short, which is done, should be done at regular and stated periods, and the habit of regularity, thus early established, would become a second nature, and prove a real blessing through life. There is not a single office, profession, or avocation, from the high duties of the Monarch down to the vile drudgery of the dustman, that does not owe half its honours, respectability, and success to punctuality."* 20.

We are rather surprised that the talented author has omitted all allusion to these habits of punctuality, which are best acquired at this early period of life.

The third chapter is on "the peculiarities of disease in infancy and childhood." It is divided into three parts—etiology, diagnosis, and prognosis.

1. *Etiology.* The great activity of the circulation, and the high susceptibility of the nervous system, predispose children strongly to acute diseases, which will, of course, be rapid in their progress and violent in their degree. But the same peculiarities confer on the organs and structures of the infant a remarkable energy and power of reparation. Irritation is easily set up in a child, and is quickly spread, from organ to organ, by sympathy.

"The most common seat of direct irritation is in some part of the alimentary canal, and this generally arises from the ingestion of unwholesome aliment. The presence of any irritating matter there gives rise to symptoms often very formidable, particularly when it occasions sympathetic disturbance in the brain. The intestinal mucous membrane itself soon takes on the irritative action, and becomes the seat of disease; the attendant symptoms generally assuming the type of remittent fever. The suffering caused by difficult dentition is a familiar example of direct irritation, which, though slight in itself, may lead to most serious results." 87.

The brain, the lungs, the liver, all sympathize with irritated digestive or-

---

* Economy of Health, p. 20.

gans in children—and, indeed, at all ages, though not so vividly or acutely as in infancy. Mental impressions, for obvious reasons, with the exception of fear and anger, are not so operative at this as at later periods of existence. Impressions of cold and damp are fertile sources of infantile illness; but our author very properly dissuades from the general use of flannel in early life. It should be reserved for ulterior stages of life.

2. *Diagnosis.* After drawing a portrait of a child in health, the author gives one of an infant in a state of disorder or disease.

"Not so with the child when ill: it is more or less peevish—dislikes being stirred, or even cries when handled; its hour of sleep is uncertain, and it rests ill, or awakes startled or crying. The skin is hot and dry, particularly of the hands, feet, and head. The flesh is soft or wasted. There is thirst, with unnatural appetite and depraved secretions.

When a sick child is brought to us, or we visit it, our attention should first be directed to the expression of the countenance, then to the attitude, state of the limbs, and skin. We next proceed to examine more minutely into the several symptoms—to determine the seat of the disorder, whether in the head, chest, or abdomen—and its nature, or the particular lesion on which it depends.

When the brows are knit, the eyes fixed and staring, or looking wild or vacant, our attention is at once directed to the head. We try whether it is unusually hot, the vessels full or in over-action, and examine the fontanelle, to see if it be depressed or in a state of distention. We now observe the attitude, and see whether the child's head hangs heavily on the nurse's arm, or rolls from side to side, sunk upon the pillow.* We examine the limbs, to see whether they be rigid or relaxed—lying motionless, or tossed about, and affected with spasm—in particular, we try whether the hands are clenched, and the thumb turned in, or toes bent. According as the upper or lower extremities are affected, one or both, we judge of the seat and extent of the disease. The state of the pupil next demands investigation. It may be found contracted, and the child will start, as if frightened, or scream out, on being touched; or the pupil may be dilated and insensible to light—the child being motionless or unconscious. We will hear that the child has had starting in its sleep, grinding of the teeth, or movements of the lips; and when awakened, it started up affrighted or screamed out; the bowels are obstinately costive, or the evacuations very foul and dark coloured. The hands are usually hot, but the feet cold; and one cheek is often deeply flushed. No doubt can now exist that the head is the seat of disease; but what that particular disease may be, and whether a primary or secondary affection, is to be determined by symptoms hereafter to be detailed in their proper place." 97.

3. *Prognosis.* In children, all acute inflammations are highly dangerous. Trismus nascentium is almost always fatal, and so are some kinds of hydrocephalus. Syphilis may always be cured, or nearly always. If a child survive the first or acute stage of a disease, there are strong grounds for hope.

### Chap. IV.—Infantile Therapeutics.

Long ago, Gaubius tried to arrange the doses of medicines according to age, and expressed in a tabular form, thus:—

---

* "Occasionally the neck is stiff or retracted."

| | | | | |
|---|---|---|---|---|
| " Dose for an adult, | .. | .. | 1 | say 1 drachm, |
| for a child 7 years old, | | .. | $\frac{1}{3}$ | — 1 scruple, |
| ——— 4 ——— | | .. | $\frac{1}{4}$ | — 15 grains, |
| ——— 3 ——— | | .. | $\frac{1}{6}$ | — 10 grains, |
| ——— 2 ——— | | .. | $\frac{1}{8}$ | — 8 grains, |
| ——— 1 and under, 1-12 to 1-15—5 or 4 grains." 106. | | | | |

On mercury, opium, and other remedies, there are many judicious observations in this chapter; but we cannot dwell on them here.

The fifth chapter is on accidents and diseases occurring at the period of birth, or shortly afterwards. This and the sixth chapter, on dentition, must also be passed over, as too elementary, and incapable of analysis. In both chapters, however, the young practitioner (perchance some of the old folks) will find much valuable information.

### CHAP. VII.—DISEASES OF THE DIGESTIVE ORGANS.

Inflammatory affections of the gastro-intestinal mucous membrane are the most common. Our author treats of the different kinds and localities—the aphthous, erythematous, exudatory, &c. We can only spare room for a short extract from this chapter—though it is one of the longest and most important in the whole work.

" A particular form of gangrene of the mouth, without any preceding inflammation, occasionally attacks infants, especially such as are very feeble at birth, or broken down by disease. An œdematous circumscribed swelling appears on the cheek, with a central point more or less hard, over which occurs a dark red spot. This spot may appear on the inside or outside of the cheek; and the skin over the œdematous part is characterised by an oily appearance. An eschar forms from within outwards on the central point; and the soft parts mortify, often extensively, down to the bone, so that the parietes of the cheeks and gums are destroyed—falling off in shreds, mixed with a dark sanguineous fluid, and accompanied by a very fetid odour.

No disease can be more frightful or formidable, than sloughing of the mouth in children. Recovery seems impossible, when once the disease has set severely in, the child sinking beneath the constitutional disturbance, independently of the local ravages of the disorder—which, however, are often such as to render recovery not to be desired, so frightful is the deformity necessarily entailed. The fever, which may be at first high, soon sinks into low typhus; the bowels become greatly deranged, and diarrhœa often attends at the close." 223.

This chapter embraces a great variety of disorders, as enteritis, iteitis, cholera infantum, remittent fever, worms, dentition diseases, &c. all of which are important to the medical practitioner.

The eighth chapter treats of diseases of the respiratory apparatus, and, like all the others, is very skilfully handled. It is too elementary, however, for an analysis. The following remarks on Dr. Ley's ingenious theory of laryngismus stridulus, may not be inopportune.

" More recently, Dr. Hugh Ley has published a lengthy essay upon spasm of the glottis, or laryngismus stridulus, as he terms it, in which he refers its cause to enlargement of the bronchial or deep cervical lymphatic glands, producing pressure upon the recurrent nerves, and consequent paralysis of the muscles supplied by them. Without going into a consideration of Dr. L.'s theories, it will be sufficient to say, that the affection is too constantly curable, and too frequently yields to a removal of specific irritations, as from dentition, deranged bowels, or indigestible aliment, to admit of its being referred to a cause so decidedly organic. That enlarged bronchial and cervical glands are frequently met with, as complications, we have no doubt; but when we recollect that the subjects of the complaint are usually of a delicate and strumous habit, such appearances are not to be looked upon as of much importance in relation to its special pathology. These remarks equally apply to the other hypotheses we have alluded to; and, therefore, we are inclined to adopt Dr. Marsh's view, and consider spasm of the glottis as essentially a spasmodic affection, but one liable to many organic complications, both accidental, and consequent upon the continuance of irritation which it occasions in the system." 359.

Our readers are aware that the above opinion corresponds with that which we have expressed in the review of Dr. Ley's very ingenious and clever work. In respect to the pathology of hooping-cough, we have the following not very consolatory statement.

"Morbid anatomy has thrown no light upon the proximate cause of hooping-cough; and accordingly, as is always the case in the absence of exact knowledge, the subject is deluged with a flood of hypotheses—none of which, however, can claim to be more than plausible guesses: many are merely a restating of ignorance, in obscure, and, therefore, according to the notion of some, scientific words. The fact is, the simple disease is probably never fatal; and, therefore, any appearances which we may meet with in the dead body are the results of complication—*e. g.* marks of inflammation in the lungs or brain." 366.

This doctrine will apply to more diseases than hooping-cough. The following short extract, respecting the treatment, contains sound advice.

"During the whole progress of a case of hooping-cough, we must be alertly upon the watch for the first signs of local inflammations. The most common of these is bronchitis, the symptoms and treatment of which are detailed under their proper head. When it does supervene or threaten, it must be met by active depletion and loss of blood, general or local, according to the symptoms; and from this practice we are not to be deterred by any preconceived ideas as to the nervous or spasmodic nature of the disease. We cannot too often repeat, that the great danger of pertussis arises from the likelihood of the supervention of local inflammations, requiring active and prompt treatment." 371.

The ninth chapter is on the exanthemata, and consists of solid elementary instruction, brought up to the level of the present state of our knowledge of the eruptive diseases. Scrofula, rickets, pemphigus, and diseases of the cerebral system, close the work.

Although our authors have had a wide field for their personal investigation of infantile diseases, yet the work is not so much characterized by *originality*, as by the judicious use which they have made of the labours of others. They have good judgment—and, by that most important instrument, they have been enabled to *compile well*—a task nearly as difficult as to *compose* well. They have woven an excellent and lasting tissue or web from yarns spun by others, and thus furnished the busy practitioner, at a small expense of money and labour, with a manual of infantile diseases, brought up to the very highest level of modern science.

